# Cortical Single-Cell Primers of Abnormal Brain Activity in Parkinson’s Disease

**DOI:** 10.34133/research.0863

**Published:** 2025-09-11

**Authors:** Daniela Mirzac, Martin B. Glaser, Svenja L. Kreis, Florian Ringel, Manuel Bange, Damian M. Herz, StanislavA. Groppa, Lilia Rotaru, Viviane Almeida, Jenny Blech, Mohammadsaleh Oshaghi, Sebastian Kunz, Matthias Klein, Jonas Paulsen, Heiko J. Luhmann, Tobias Bopp, Philip L. de Jager, Sergiu Groppa, Gabriel Gonzalez-Escamilla

**Affiliations:** ^1^ Department of Neurology, Saarland University, Homburg, Germany.; ^2^ Department of Neurology nr. 2, Nicolae Testemitanu State University of Medicine and Pharmacy, Chisinau, Republic of Moldova.; ^3^ Institute of Physiology, University Medical Center of the Johannes Gutenberg University Mainz, Mainz, Germany.; ^4^ Neuroimaging Center, University Medical Center of the Johannes Gutenberg University Mainz, Mainz, Germany.; ^5^ Department of Neurosurgery, University Medical Center of the Johannes Gutenberg University Mainz, Mainz, Germany.; ^6^ Neurology and Neurosurgery Institute “Diomid Gherman”, Chisinau, Republic of Moldova.; ^7^ Department of Biosciences, Faculty of Mathematics and Natural Sciences and Centre for Bioinformatics, Department of Informatics, University of Oslo, Oslo, Norway.; ^8^ Institute of Immunology, Research Center for Immunotherapy (FZI), University Medical Center of the Johannes Gutenberg University Mainz, Mainz, Germany.; ^9^ Center for Translational & Computational Neuroimmunology, Department of Neurology and the Taub Institute for Research on Alzheimer’s Disease and the Aging Brain, Columbia University Irving Medical Center, New York, NY, USA.

## Abstract

Abnormal brain oscillatory activity is a well-established hallmark of bradykinesia and motor impairment in Parkinson’s disease (PD), yet its molecular underpinnings remain unclear. To address this gap, we analyzed over 100,000 single-cell RNA transcriptomes from fresh dorsolateral prefrontal cortex tissue of individuals with PD and non-PD controls, undergoing deep brain stimulation—2 cohorts, which open up an unprecedent window to the characterization of human cortical brain tissue, aiming to uncover the molecular mechanisms of abnormal brain oscillatory activity in PD. Fresh brain tissue samples offer a unique opportunity to precisely elucidate the molecular underpinnings of known, clinically relevant electrophysiological hallmarks of neurodegeneration, which can be used to inform targeted therapeutic strategies. We depicted in microglia and astrocytes enrichment of mitochondrial electron transport and oxidative phosphorylation pathways, which were directly linked to the increase of pathological brain activity and the decrease of prokinetic brain activity. Additionally, the abnormal phase–amplitude coupling of beta–gamma brain activity was related to the dysfunction of oligodendrocyte precursor cells and inflammasome activation mediated by lymphocyte-driven adaptive immunity. We identified a distinct set of dysregulated genes from the mitogen-activated protein kinase phosphorylation pathways, mitochondrial electron transport at the intersection of neuroinflammation and neurodegeneration, suggesting pivotal roles in PD pathology. This unique dataset provides unprecedented insights into the immune and metabolic dysregulation underlying PD, offering a mechanistic framework for understanding invasive transcriptomic biomarkers related to prokinetic and pathologic brain activity in PD.

## Introduction

Parkinson’s disease (PD) is the second most common neurodegenerative disorder, characterized by dopaminergic neuron loss along with the formation of intraneuronal α-synuclein inclusions called Lewy bodies [1]. In PD, neuronal cell loss leads to altered neurotransmitter signaling and dysfunction of excitation–inhibition balance, thus triggering abnormal patterns of action potentials, synaptic dysregulation, and pathologic oscillatory activity in widespread brain circuits [2]. The motor symptoms of PD are linked to abnormal synchronization of the basal ganglia-thalamo-cortical circuits, which converge on the primary motor (M1) and the premotor cortex (PMC) [3]. Pathophysiological oscillatory activity, particularly increased beta and reduced gamma activity [4–6], is regarded as an electrophysiological hallmark of bradykinesia and rigidity in PD [7,8]. Increased cortical beta activity further reflects impaired cognitive top-down control, such as in motor inhibition [9], while reduced gamma frequency oscillations in the prefrontal cortex and PMC associate with exacerbated movement symptoms in PD [10]. Growing body of evidence indicates that the coupling between the phases and amplitudes (PAC) of this pathological oscillatory activity may serve as possible mechanism of disrupted brain network dynamics in PD [11]. Particularly, increased beta–gamma PAC in regions involved in motor control, including the sensory motor cortices, has been consistently reported to be associated with the severity of the motor symptoms [12,13]. Yet, the molecular mechanisms underlying abnormal pathophysiological oscillatory activity remain unknown.

Recently, a tight interrelation between the pathological cortical brain activity in PD and immunometabolic dysregulation has been shown in PD patients [14]. In this work, the authors suggest that abnormal cell types and their gene expression may be targeted through therapeutic interventions with direct impact to pathological cortical brain activity. Accordingly, it has been suggested that beta oscillations are related to an altered redox environment or have an abnormal sensitivity to superoxide redox parameters [15]. Gamma oscillations are associated to the disruption of central nervous system (CNS) homeostasis, which may be regulated via diverse functions of microglia, specifically immune responses and metabolic pathways [16]. Thus, glial cells, including microglia, play a pivotal role in neuroinflammation, which in turn exacerbates disease progression over time and may accelerate apoptosis through the intrinsic mitochondrial pathway [17,18].

Previous studies have attempted to assess the molecular underpinnings of PD pathology using postmortem brain samples, particularly from the substantia nigra [19–21], whereas other studies on living participants have aimed at noninvasively or minimally invasively track disease progression [21]. However, the transcriptomic profiles from postmortem data present an altered molecular landscape due to RNA/protein degradation after death, while peripheral markers contain cell types and molecules that may not cross into the brain, thus only partially mirroring CNS immune profiles and often diverging in signature and behavior, leaving unanswered how real-time, dynamic cellular and molecular interactions occur in the brain.

To fill this gap, while providing deeper evidence on the mechanisms of abnormal oscillatory activity in PD, we leverage single-cell RNA sequencing (scRNA-seq) from fresh dorsolateral prefrontal cortex (DLPFC) from living patients, offering a timely and unparalleled opportunity to link cell-specific transcriptomics with clinically relevant electrophysiological phenotypes. Further, we provide cell type-specific biological relevance, as well as potential mechanisms driven by targetable genes. Our data-driven framework may serve as basis for detailed characterizations of in vivo pathophysiology and deliver more reliable biomarkers for neurodegeneration.

## Results

### Broad cell type composition of PD and non-PD

**Fig. 1.**
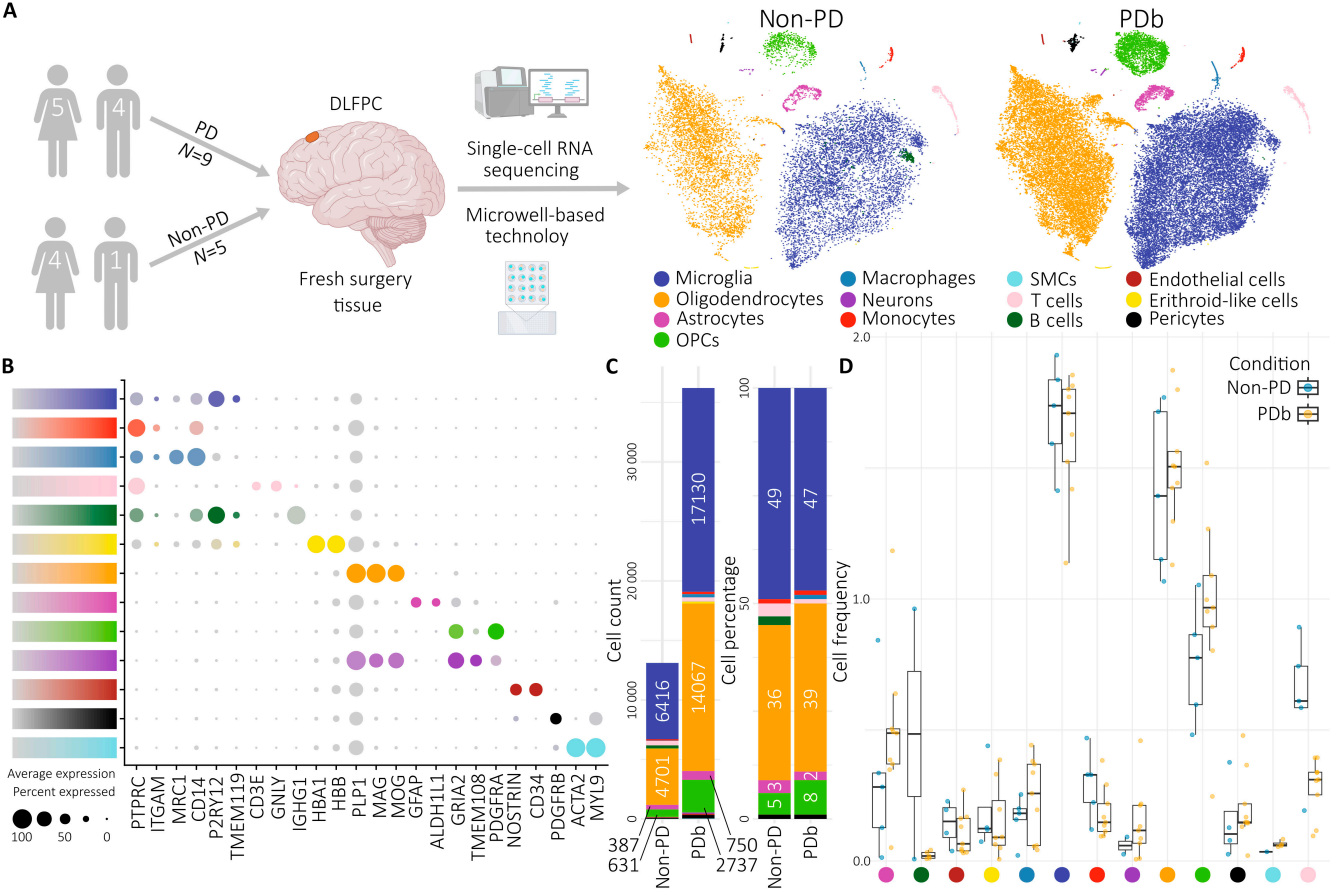
Overview of the experimental approach and scRNA-seq data. (A) Workflow for the generation of the scRNA-seq dataset [14]. Experimental approach from surgical retrieval of DLPFC samples to fresh tissue processing, single-cell isolation, and data analysis. t-SNE map visualization of the cell clusters of 9 PD and 5 non-PD samples integrated in the downstream analysis. This panel was partially created with BioRender.com. (B) Cell types annotation according to expression of known marker genes [82]. Average expression colored by corresponding cell type. (C) Stacked bar plots depicting cell count and cell percentage distributions of all cell types across cohorts of PDb and non-PD subjects. (D) Box plot of cell frequency of all cell types across individual patients. Labeled by cohort and cell type. SMCs, smooth muscle cell; PDb, Pakrinson's disease patients with brain biopsies; Non-PD, patients without PD.

To characterize the molecular single-cell gene expression signatures of PD in vivo, we studied a novel scRNA-seq dataset from fresh DLPFC tissue from patients who underwent deep brain stimulation (DBS) surgery [14]. The dataset includes 101,691 RNA transcriptomes across 2 groups: 9 PD subjects and 5 non-PD subjects (Fig. 1A). After filtering and quality control, we retained 49,330 RNA transcriptomes for the downstream analysis, with an average of 3,500 cells per subject. This amounted to 36,216 cells for the PD cohort and 13,114 cells for the non-PD cohort (Fig. 1C).

Following cross-sample alignment and graph-based clustering, all sequencing data were integrated and represented according to their spatial arrangements, independently to their donor (Fig. 1A and Methods). This resulted in 11 distinct cell types with specific cell type marker expression (Fig. 1B and Methods). The majority of cell populations in our data consisted of glial cells. In order of frequency, we observed microglia 47% in the PDb (patients with PD and brain biopsies) and 49% in the non-PD, oligodendrocytes 39% and 36%, OPCs (oligodendrocyte precursor cells) 8% and 5%, and astrocytes 2% and 3%, respectively (Fig. 1C). To minimize the influence of cell variability, the present dataset employs highly matched biological replicates to reduce background and technical noise. Therefore, there were no significant group differences in cell frequency for each cell type (Fig. 1D).

### Cell type-specific transcriptome profiling identifies metabolic and inflammatory pathways dysregulated in PD compared with non-PD

Emerging evidence indicates the implication of astrocytes, microglia, oligodendrocytes, and oligodendrocyte progenitor cells in PD pathogenesis [19,22–25]. Thus, we further selected the most prevalent cell types in our dataset (*n* = 4) to explore their molecular profiles. We performed differential gene expression analysis, followed by unbiased gene set enrichment analysis (see Methods), identifying differently enriched and depleted pathways.

**Fig. 2.**
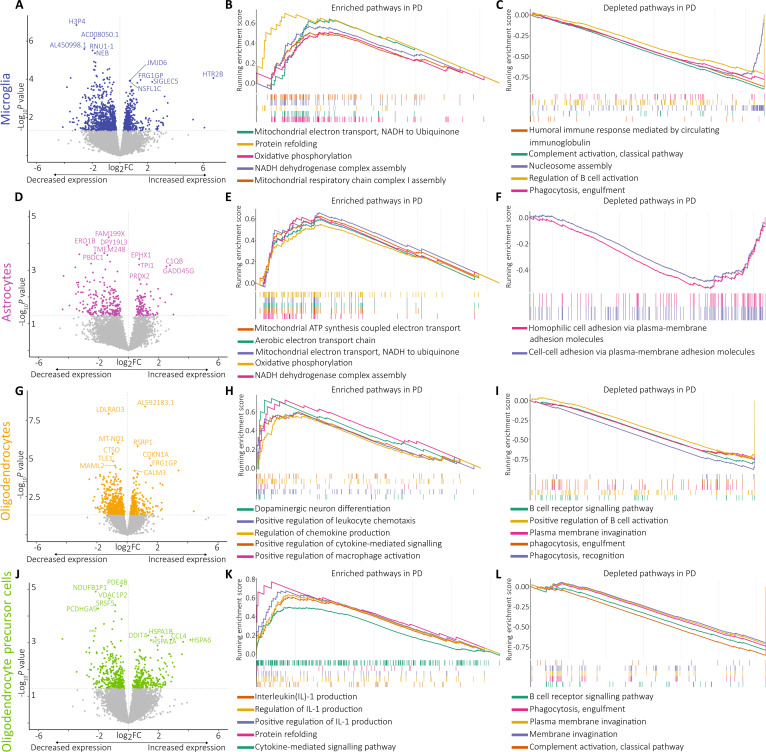
Transcriptomic analysis of human cortical cell types (microglia, astrocytes, oligodendrocytes, and oligodendrocyte progenitor cells). (A) Volcano plot of the differentially expressed genes in microglia cell type in PD versus non-PD group [83]. (B) Gene set enrichment analysis (GSEA) plots of the gene ontology biological processes (GOBP) pathways enriched in microglia cell type [84]. Top 5 terms with positive normalized enrichment score (NES) are shown. (C) Top 5 terms with negative NES shown. (D) Volcano plot of the differentially expressed genes in astrocytes in PD versus non-PD group. (E) GSEA plots of the GOBP pathways enriched in OPC cell type. Top 5 terms with positive NES shown. (F) Top 2 terms with negative NES shown. (G) Volcano plot of the differentially expressed genes in oligodendrocytes cell type in PD versus non-PD group. (H) GSEA plots of the GOBP pathways enriched in astrocyte cell type. Top 5 terms with positive NES shown. (I) Top 5 terms with negative NES shown. (J) Volcano plot of the differentially expressed genes in OPC-type in PD versus non-PD group. (K) GSEA plots of the GOBP pathways enriched in astrocyte cell type. Top 5 terms with positive NES shown. (L) Top 5 terms with negative NES shown.

In microglia, the resident immune cells in the brain that function as the neural tissue’s defense system and contribute to the development and maintenance of neural circuits [26], we identified 34 up-regulated and 40 down-regulated pathways in PDb compared to non-PD (Fig. 2A and B). Our findings are consistent with the general consensus of significant dysregulation of mitochondrial pathways in PDb [27–30]. However, the results differ to the depleted mitochondrial-related pathways found in particular regions such as the caudate and putamen [31], thus suggesting region-specific dysregulations. Furthermore, we observed abnormal pathways supporting metabolic dysregulation in microglia (Fig. 2A to C). The key dysregulated genes in PDb, namely, HSP90AA1, HSPA1A, HSPD1, and DNAJA4, are involved in folding and misfolding of proteins.

Misfolded proteins may not only disrupt mitochondrial function and endocytosis, functions also affected in our patients, but also potentially modulate innate immune responses [17]. Pathways related to innate immune responses directly observed in our data were related to humoral immune response, and complement activation through the classical pathway (Fig. 2A and C) and to genome architecture and regulation was also down-regulated (Fig. 2A and C). The former is consistent with previous studies showing dysregulated inflammation-related pathways in PD compared to non-PD [32]. The latter results corroborate previous findings of a PD-associated gene expression regulation system [33].

Despite previously being considered to be passive cells, current evidence sets astrocytes as active contributors of brain homeostasis [34]. Microglia–astrocyte interactions represent a delicate balance exhibiting altered gene expression profiles that are predicted to affect their function [35], and in our dataset, the disease-specific transcriptome changes in the astrocytes were similar to those observed in microglia. We identified 25 enriched and 2 depleted gene ontology terms for biological processes (GO BP) terms (Fig. 2D to F), corroborating previous studies showing altered vesicle handling and synaptic vesicle dynamics in PD [36–38]. Moreover, further pathways were involved in antigen presentation via major histocompatibility complex (MHC) class II complexes, suggesting that astrocytes may function as antigen-presenting cells in PD, as shown in cell culture studies [39], and become reactive adopting a pro-inflammatory phenotype in response to activated microglia [40,41].

In our study, we identified similar and concordant transcriptome abnormalities in oligodendrocytes and OPCs, when comparing PDb and non-PD. For oligodendrocytes, we identified 84 enriched and 20 depleted GO BP terms, and for OPCs, we identified 388 enriched and 39 depleted GO BP terms. In both cell types, we reported a cluster of dysregulated immune and metabolic pathways, as we described above in microglia and astrocytes (Fig. 2G, I, J, and L). Unlike other cell types, both oligodendrocytes and OPCs presented a cluster of enriched pathways related to immune activation and cytokine signaling (Fig. 2G, H, J, and K). These findings align with cytokine signaling and stress response to unfolded protein pathways, indicating the participation of these 2 glial cell types in the neuroinflammatory process [20,42]. The enrichment of interleukin-1 (IL-1) pathway in OPCs reflects an inflammatory state of PDb compared with non-PD and is consistent with literature findings [43]. Recent reviews explore the further implications of these findings with other clinical indicators and at the peripheral level [43,44].

Within observed altered pathways in OPCs, key dysregulated genes, P2RX7 and PRKCB, are involved in neuroinflammatory processes. Specific only for oligodendrocytes was the enrichment of the “dopaminergic neuron differentiation” pathway (Fig. 2H), which is in line with the respective cell function, and has already been reported as an altered pathway in PD [36]. Similar to the microglia, in OPCs, we attested abnormal protein refolding (Fig. 2K).

### Cell type-specific gene coexpression correlates of abnormal brain oscillatory activity

**Fig. 3.**
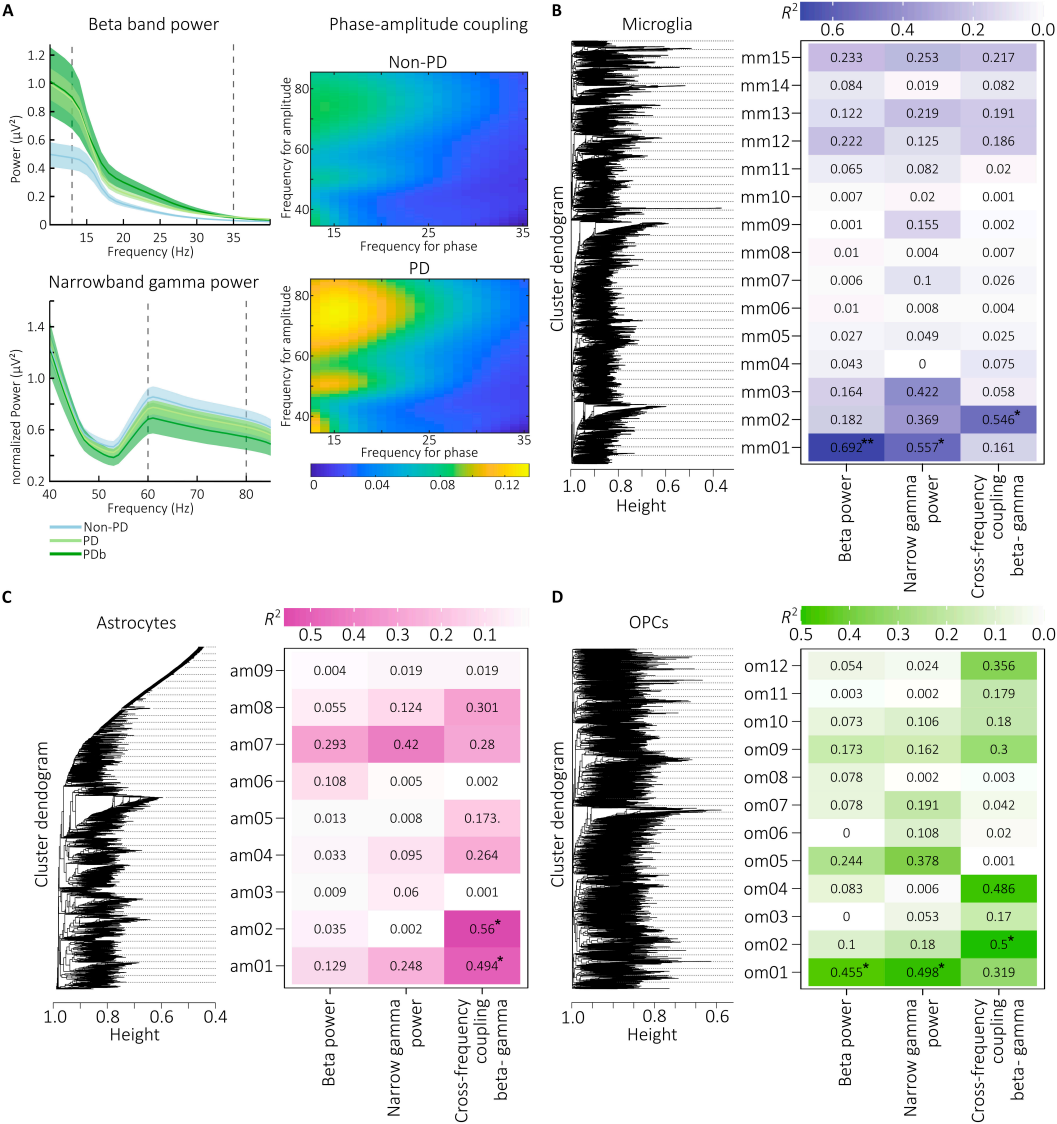
Electropathophysiological hallmarks of PD associated with cell type-specific modules of genes. (A) Spectral power for frequencies ranging from 1 to 40 Hz (top left) and normalized power from frequencies ranging from 40 to 85 Hz (bottom left). The vertical dotted lines mark the ranges of the beta and narrow gamma bands. Patients with PD (green) present increased cortical beta and reduced cortical narrowband gamma power in comparison to non-PD (blue). The spectral plots (right) show increased cortical phase–amplitude coupling between the phase of beta and the amplitude of gamma power in PD compared to non-PD. (B) Correlations between WGCNA microglia modules and EEG hallmarks. *R*^2^ value of the correlation for each comparison shown in the table. Results are colored by *R*^2^ value. Statistically significant results are shown with asterisk. (C) Correlations between WGCNA astrocyte modules and hallmark data. *R*^2^ value of the correlation for each comparison shown. Results are colored by *R*^2^ value. Statistically significant results are shown with asterisk. (D) Correlations between WGCNA OPC-type modules and EEG hallmarks. *R*^2^ value of the correlation for each comparison shown. Results are colored by *R*^2^ value. Statistically significant results are shown with asterisk. *P ≤ 0.05; **P ≤ 0.01; ***P ≤ 0.001.

To validate the robustness of the observed oscillatory activity abnormalities in PDb, a large group of 91 PD patients was recruited and contrasted against 38 age- and sex-matched healthy controls. PD and PDb had identical electrophysiological hallmarks (Fig. 3A) as compared to controls for bradykinesia hallmarks (PD versus control: *T* = 2.77, *P* = 0.003; PDb versus control: *T* = 2.68, *P* = 0.005) and rigidity hallmarks (PD versus control: *T* = 1.8, *P* = 0.037; PDb versus control: *T* = 2.34, *P* = 0.012). PAC abnormalities were also found for PD versus control (*T* = 1.81, *P* = 0.036) and marginally significant in PDb (*T* = 1, *P* = 0.05). The replication of bradykinesia and rigidity hallmarks and the consistent directionality of the trending PAC abnormalities suggest that the PDb cohort captures the same biological features despite the reduced statistical power, altogether matching previous reports of abnormal brain activity in PD [4–6,8]. We then investigated the multifactorial association [through weighted gene coexpression network analysis (WGCNA)] of pathological oscillatory activity with molecular features combined in distinct modules for each cell type.

In microglia, 2 (out of 15 total) individual modules correlated with the pathological brain activity—module 1 (containing 969 genes) and module 2 (212 genes), whereas in OPCs, 2 (out of 12 total) modules emerged—module 1 (2,393 genes) and module 2 (3,200 genes) (Fig. 3B and D). From these, microglia module 1 and OPC module 1 exclusively correlated with the motor symptom hallmarks but not with their coupling, suggesting that the abnormal oscillatory activity could mainly reflect molecular changes in microglia and OPCs. The analysis on oligodendrocytes did not reveal any associated modules (results not shown). In astrocytes, 2 (out of 9 total) individual modules correlated with PAC (Fig. 3C).

Overall, abnormal oscillatory activity coupling was associated with microglia, OPCs, and astrocytes, evidencing a common and cohesive involvement [20] as the molecular mechanism underlying the PAC, specifically affecting metabolic regulation in microglia and OPCs, with inflammasome involvement.

### Biological substrate driving pathophysiological brain activity

To attest biological meaning to the identified gene modules, we evaluated the association between their molecular activity (e.g., pathway dysregulations in PDb as compared to non-PD) with abnormal brain activity.

**Fig. 4.**
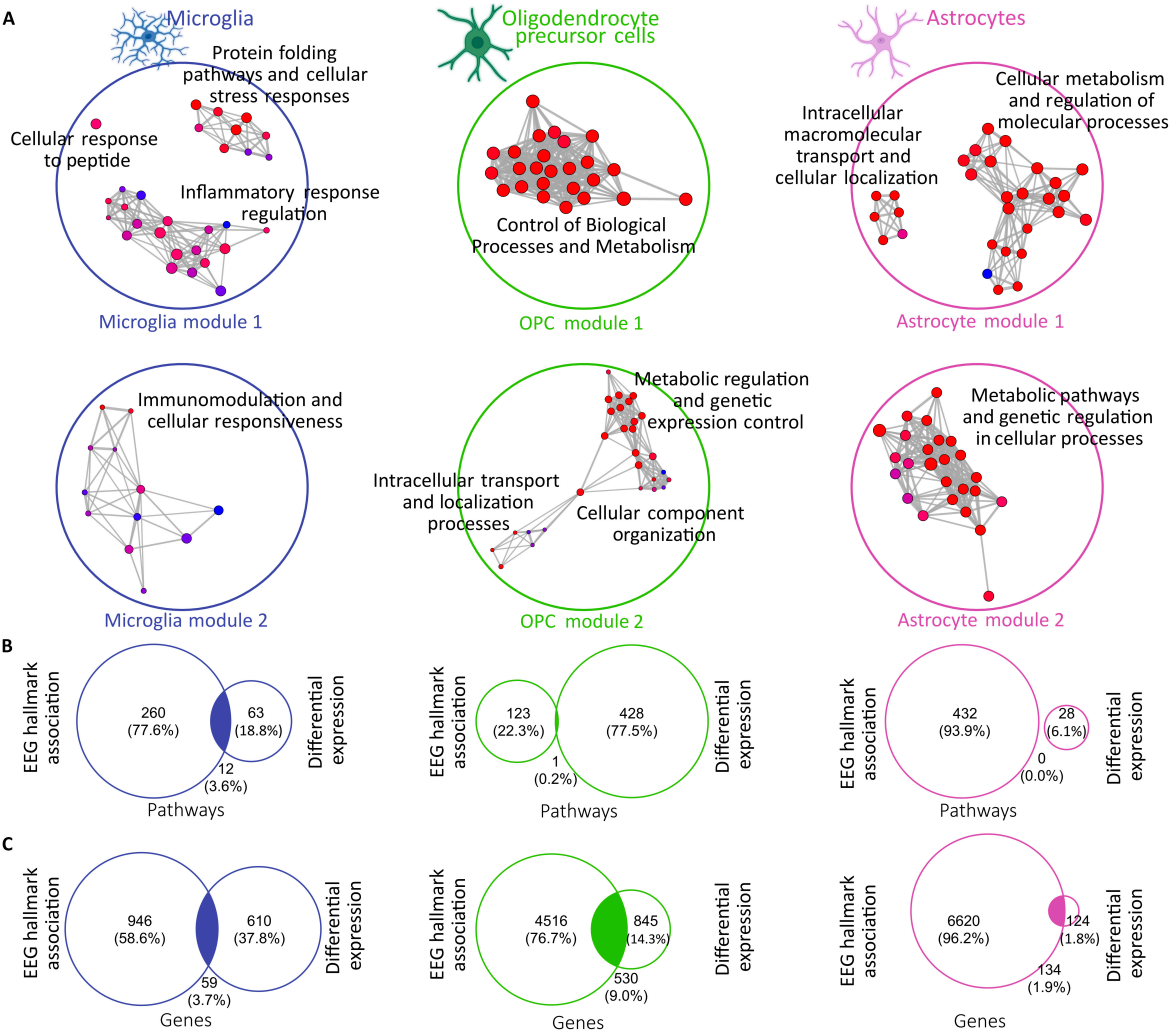
Cell type-specific biological relevance for clinically established EEG hallmarks. (A) Enrichment map for overrepresentation analysis on gene ontology pathways as provided by PANTHER [86] for relevant WGCNA modules: microglia in blue, OPCs in green, and astrocytes in pink. Size of the circle corresponds to the count of genes in each term, color-coded by FDR, filtered only for statistically significant results (FDR < 0.05). (B) Overlap between the pathways enriched with gene set enrichment analysis versus overrepresentation analysis for each cell type. (C) Overlap between the genes enriched with gene set enrichment analysis versus overrepresentation analysis for each cell type.

Microglia module 1, correlated with beta (*R*^2^ = 0.692, *P* = 0.005) and gamma power (*R*^2^ = 0.557, *P* = 0.021), consisted of 263 BP and 33 molecular function (MF) overrepresented terms (Fig. 4A). OPC module 1, correlated with bradykinesia-related (*R*^2^ = 0.455, *P* = 0.046) and rigidity-related hallmark activity (*R*^2^ = 0.498, *P* = 0.034), consisted of 55 BP and 42 MF overrepresented terms (Fig. 4A). Microglia module 2, correlated with PAC (*R*^2^ = 0.546, *P* = 0.023), comprised 12 BP overrepresented terms. OPC module 2 (*R*^2^ = 0.5, *P* = 0.033) comprised 55 BP and 27 MF overrepresented terms. Astrocyte module 1 (5,146 genes; *R*^2^ = 0.494, *P* = 0.035) comprised 55 BP and 59 MF overrepresented terms. Astrocyte module 2 (2,093 genes; *R*^2^ = 0.56, *P* = 0.02) comprised 56 BP and 43 MF overrepresented terms (Fig. 4A).

As the transcriptome profiling (Fig. 2) and the correlation (Fig. 3) analyses were performed independently, we then overlapped the resulting genes, revealing genes and pathways dysregulated in PD, directly correlating with the abnormal oscillatory patterns (Fig. 4B and C; see Methods).

### Putative genes of abnormal brain oscillatory activity in PD

Further, we looked for the overlap between the genes from the modules in each cell type and the corresponding overrepresented genes from the comparison between PDb and non-PD. This method ensures that we only explore relevant dysregulated genes in the context of the modules when assessing protein–protein interactions in STRING. In microglia, we report an overlap of 59 genes; in OPCs, we report an overlap of 530 genes; and in astrocytes, we report an overlap of 134 genes (Fig. 4C).

**Fig. 5.**
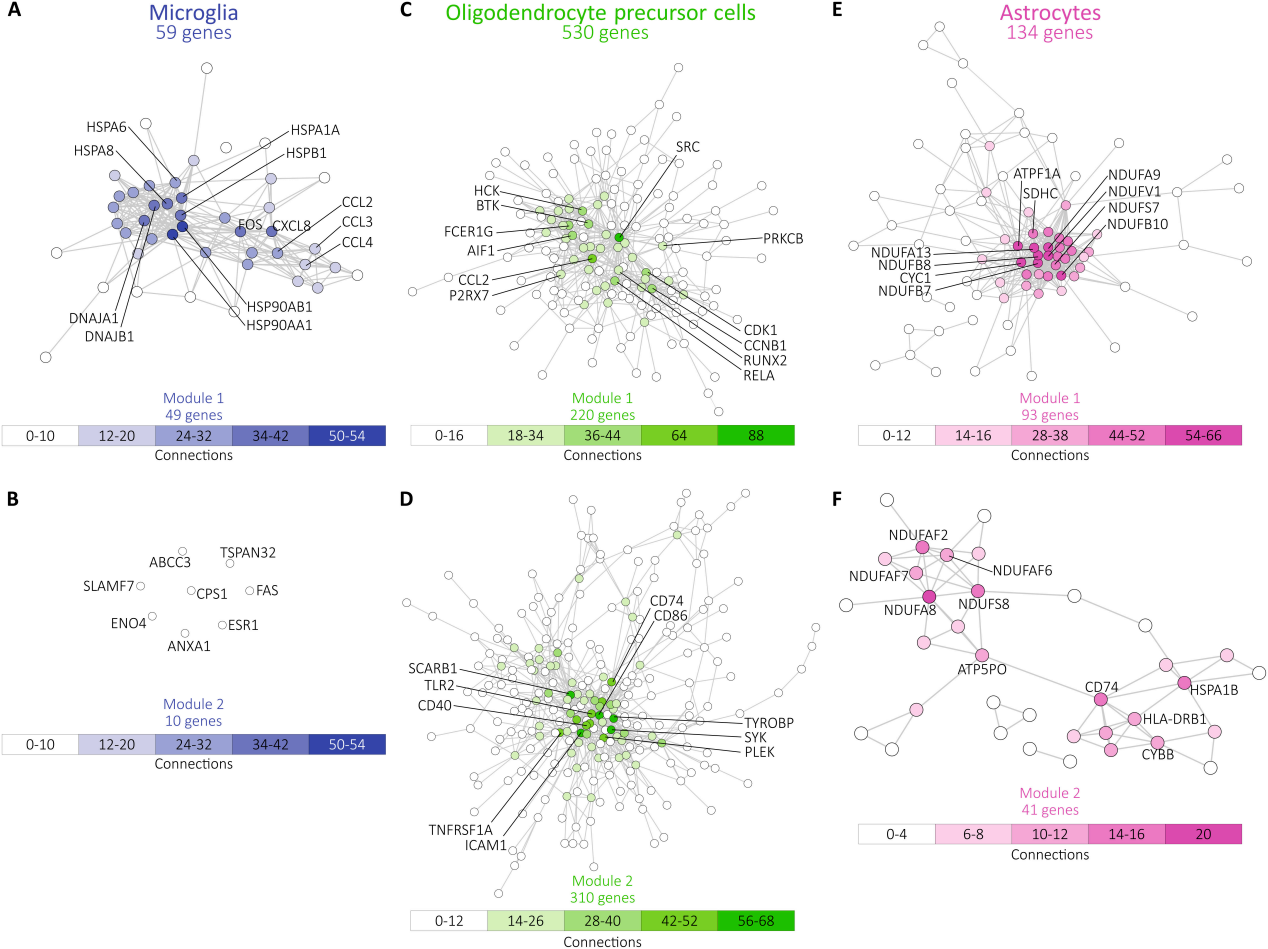
Protein–protein interaction networks of dysregulated genes. (A) Network of protein–protein interaction of dysregulated genes included in microglia module 1 (in blue). (B) Network of protein–protein interaction of dysregulated genes included in microglia module 2 (in blue). (C) Network of protein–protein interaction of dysregulated genes included in OPC module 1 (in green). (D) Network of protein–protein interaction of dysregulated genes included in OPC module 2 (in green). (E) Network of protein–protein interaction of dysregulated genes included in astrocyte module 1 (in pink). (F) Network of protein–protein interaction of dysregulated genes included in astrocyte module 1 (in pink). Genes colored by rank according to the number connections in STRING [87].

Within microglia, 49 genes are attributed to module 1 and 10 genes to module 2. When analyzing the core driver genes of module 1 (Fig. 5A), we highlight 2 directions—one dysmetabolic (e.g., HSP90AA1, DNAJA4, HSPA1A, and HSPD1) and one inflammatory (e.g., CCL2, CCL3, CCL4, and CXCL8). In module 2 (Fig. 5B), not all genes interacted with each other, resulting in a single functionally significant gene set. In the OPCs, the overlap analysis resulted in a higher number of relevant genes: 220 in module 1 (Fig. 5C) and 310 in module 2 (Fig. 5D). In both modules, the core drivers are genes related to the inflammasome—CCL2, P2RX7, and PRKCB in module 1 and CD74, CD86, CD40, and ICAM1 in module 2. Within the astrocyte cell type, we highlight 93 genes in module 1 (Fig. 5E) and 41 genes in module 2 (Fig. 5F). The genes in module 1 (NDUF family, SDHC, and ATPF1A) encode subunits or assembly factors of mitochondrial complexes I, II, and V, which are essential for oxidative phosphorylation and adenosine triphosphate (ATP) production [45], thus revealing core components of cellular metabolism and mitochondrial function. Module 2 includes genes involved in antigen presentation and immune signaling (CD74 and HLADRB1), oxidative stress and inflammation (CYBB and HSPA1B), and mitochondrial complex I assembly and function (NDUFS8, NDUFA8, NDUFAF6, and NDUFAF2). These suggest a link between disrupted metabolism, immune dysfunction, and disease progression.

Next, we performed an intersection between the gene sets (GO terms) overrepresented in the modules with the terms overrepresented in the transcriptomic comparison between PDb and non-PD. While there was no overlap in the astrocytes, there was one in microglia and OPCs of 12 terms and 1 term, respectively (Fig. 4B). Thus, we can infer that the abnormal rhythmic oscillations in PD may reflect dysregulations guided by microglia and OPCs.

**Fig. 6.**
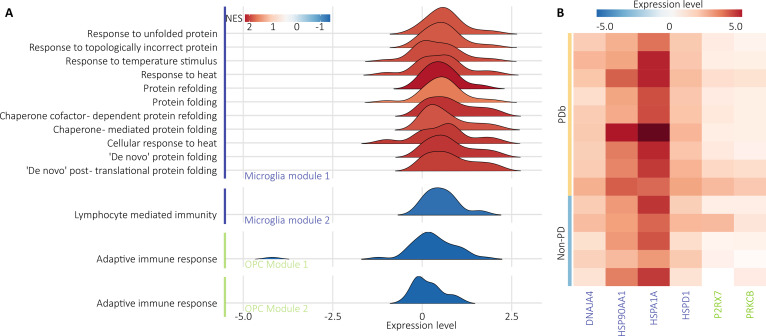
Pathway directionality and gene expression. (A) Ridgeplot visualization of the overlapping pathways for microglia (in blue) and OPCs (in green). Pathways with corresponding genes from the overlapping analysis between the differential transcriptome profiling analysis and correlation analysis with the electropathophysiological hallmarks. Normalized enriched score from the differential transcriptome profiling analysis [84]. Gene expression values for each individual gene from the differential gene expression analysis [83]. (B) Heatmap of the expression level from downstream analysis [81] of relevant genes aggregated at the patient level.

The overlapping pathways related to metabolic dysregulation, such as “protein refolding” and “heat shock response” were overrepresented, whereas the pathways related to immunity were underrepresented, regardless of cell type (Fig. 6A).

In microglia, module 1 more strongly associated with abnormal brain activity, evidencing impaired metabolism as a possible molecular basis for antikinetic activity (Fig. 6A). The differential gene expression identified 4 up-regulated genes in PDb compared to non-PD: HSP90AA1, HSPA1A, HSPD1, and DNAJA4 (Fig. 6B).

In microglia module 2 and OPC module 2, which mainly correlated with PAC, altered pathways of immune response emerged (Fig. 6A), evidencing that abnormal brain oscillations related to motor dysfunction are susceptible to the disruption of microglia activity, involving depletion of immune pathways. We have identified 2 up-regulated genes in OPCs in PDb compared to non-PD: P2RX7 and PRKCB (Fig. 6B). Identification of these pathway-specific genes unveils a novel spectrum of molecular targets, offering a strategic entry point for modulating disease pathology.

## Discussion

In the present study, we demonstrate that the pathological increase in beta power and the decrease in gamma power, as electrophysiological hallmarks of PD, are tightly related with dysregulated molecular pathways. More specifically, transcriptional changes in microglia, astrocytes, OPCs, and oligodendrocytes highlighted key involvement of metabolic and immune pathways. The dysregulated pathways in microglia and astrocytes were related to protein homeostasis and metabolism, while in OPCs, oligodendrocytes and astrocytes were related to the inflammasome pathways. The common involvement of multiple glial cell types provides evidence for a common link between neuroinflammation and neurodegeneration mediated through metabolic pathways, which converge on a small number of key genes.

Beta frequency oscillations, recognized as antikinetic in PD, are proposed to be modulated by the redox environment and to be sensitive to superoxide redox parameters [15]. Gamma frequency oscillations, considered to be prokinetic in PD, are reported susceptible to the disruption of CNS homeostasis, which may be associated with diverse functions of microglia, not only immune response and metabolic pathways but also converging on mitochondrial reactive oxygen species (ROS) synthesis [16]. From the main up-regulated genes in our study, HSPD1, P2RX7, and PRKCB are implicated in dysregulated mitochondrial metabolism as follows. P2RX7 is a member of the P2X family, known to have up-regulated expression in microglia, astrocytes, oligodendrocytes, and OPCs, under neuroinflammatory conditions [46]. PRKCB is localized at the mitochondrial level, being implicated in the regulation of mitochondrial integrity, oxidative phosphorylation, hypoxic stress, and vascular dysfunction and triggering mitogen-activated protein kinase (MAPK) phosphorylation pathways [47]. Increased levels in heat shock protein family D (HSPD), as well as the other previously mentioned chaperones HSP90AA1 and HSPA1A, are known to be associated with PD [20,21,48,49].

Particularly, HSPD1 is involved in protein folding within mitochondria [50]. Mitochondrial protein dysfunction leads to excessive oxidative stress and cell damage, processes that are correlated with PD [14]. P2RX7 are ion-gated channels activated by ATP [51]. P2X receptors promote exchange of cations, mainly Ca^2+^, Na^+^, Mg^2+^, K^+^, and Ca^2+^ induced intracellular pathways. These channel receptors are key elements for the communication between neuronal and glial cells and establish a direct link between pathological brain oscillatory activity and cell metabolism. Indeed, under increased ATP conditions, activation of P2RX7 leads to the already discussed neuroinflammatory changes. PRKCB inhibits autophagy by negatively modulating the mitochondrial homeostasis [52]. The fragmentation of dysfunctional mitochondria, which precedes autophagy, is modulated by a specific ubiquitin ligase, PARK2, and its interaction with the kinase PINK1 [53]. Following the accumulation of PINK1, the consequent induction of PARK2 stabilization initiates mitochondrion engulfment [52]. Importantly, not only dysregulations of PINK1 and PARK2 are relevant in PD pathophysiology, but also mutations within these genes are directly linked to early-onset PD [54].

Indeed, by altering the cell energy level, mitochondrial metabolism plays a critical role in the pathogenesis of neurodegenerative disorders such as PD [55]. Our data confirm previous suggestions [45,56] of immunometabolism as the potential key determinant of cell type-specific molecular dysregulation at the interface between neuroinflammation and neurodegeneration [57]. Accumulating evidence demonstrates that α-synuclein pathology directly contributes to mitochondrial dysfunction through several independent mechanisms. These include inhibition of complex I, disruption of mitochondrial protein import via TOM20, interference with ATP synthase and mitochondrial permeability transition pore opening, and dysregulated calcium exchange due to loosened mitochondria contacts [56]. Collectively, these mechanisms converge on a shared pathological phenotype of elevated oxidative stress, impaired mitochondrial membrane potential, and reduced mitochondrial respiration. Specifically, dysregulation in PD-related genes such as PRKN and LRRK2 contributes to mitochondrial dysfunction through distinct but converging mechanisms: Parkin (PRKN) regulates mitophagy and inflammation [58], while LRRK2 disrupts mitophagy, mitochondrial membrane potential, and degradation in a cell type-specific manner [59]. Additionally, targeting neuroinflammation via the kynurenine pathway yields protective effects on mitochondrial function, oxidative stress, and dopaminergic signaling in a 6-hidroxidopamina (6-OHDA)-induced PD mouse model [60], supporting mitochondrial driven immune-metabolic modulation as a therapeutic strategy in PD.

Neuroinflammation is also associated with pathophysiological brain activity. Dysregulated lymphocyte-mediated immunity and the involvement of adaptive immune responses in PD [17,61] correlate with increased PAC. Within these pathways, we reported on the up-regulation of the following genes: HSPD1, P2RX7, and PRKCB. Heat shock proteins related to HSP90 are known to regulate inflammatory processes, including the cellular damage-related P2X7R/NLRP3 inflammasome and the autoproteolytic activation of caspase-1, which ultimately leads to secretion of the pro-inflammatory cytokine IL-1β. In our study, we observed differences in gene expression involving IL-1 pathway activation in PDb as compared to non-PD, potentially reflecting underlying inflammatory mechanisms relevant to PD. Future studies with matched cytokine profiling will be essential to further validate these observations.

HSPD1 was shown to have a role in an anti-neuroinflammatory response through microglial activation [49]. HSPD1 up-regulation was reported in the substantia nigra and striatum, regions included in the basal ganglia thalamic circuits [3], which we now expand to the cortical level.

P2RX7 was shown to regulate the activation and proliferation of microglia, directly contributing to neuroinflammation through microglia-mediated neuronal death, glutamate-mediated excitotoxicity, and inflammasome activation that results in initiation, maturity, and release of the pro-inflammatory cytokines and generation of ROS and nitrogen species [62]. P2RX7-induced microglia activation has been detected in PD [63]. In the brains of subjects with PD, α-synuclein binding and activating P2RX7 on microglia has been described [64,65]. Our findings corroborate the hypothesis that microglial hyperactivation and subsequent neuroinflammation are concomitant during neurodegeneration [51]. In a rat model of PD, in which increased microglial activation was accompanied by P2RX7 overexpression, P2RX7 antagonists promote neuroregeneration via reduced microglial activation [66]. Consequently, blocking P2RX7 in hemiparkinsonian rats reduced dopamine-induced dyskinesia and motor incoordination [67].

Thus, P2RX7 modulation is a promising option for treatment of neurodegenerative diseases [46]. However, despite its apparent efficacy in preclinical studies, translating these findings into human trials faces several challenges. For example, achieving sufficient blood–brain barrier (BBB) penetration, since many early compounds were designed for peripheral use, they fail to cross into the CNS [68]. Another major challenge involves species-specific differences, as many antagonists show potent activity at human P2X7R but poor efficacy in rodents, hindering in vivo validation [69]. Furthermore, some compounds like Brilliant Blue G suffer from nonspecificity, while others such as CE-224535 and GSK-1482160 show limited rodent receptor affinity despite promising human-targeted results [68–70]. Nevertheless, newer brain-penetrant compounds like JNJ-54175446 and JNJ-55308942 have progressed to phase II trials, indicating advances in overcoming pharmacokinetic and translational barriers [68,69]. Further drugs with anti-inflammatory properties targeting modulation of oxidative stress, mitochondrial dysfunction, and neuroinflammation might be of interest [71,72].

Similarly, PRKCB was shown to promote increased infiltration of immune cells [61]. PRKCB overexpression, as seen in our results, has been previously reported in advanced neurodegenerative stages [i.e., PD and Alzheimer’s disease (AD)] [73]. Conversely, underexpression of PRKCB leads to severe immunodeficiency [74].

Furthermore, neurodegeneration is associated with pathophysiological brain activity. Protein folding and refolding, chaperone-mediated protein folding, and response to unfolded protein have been described in PD [20,42]. These dysregulated pathways have a strong association to increased beta and reduced gamma power. We reported on the up-regulation of HSP90AA1, HSPA1A, HSPD1, and DNAJA4 within the enriched metabolic pathways in PD. HSP90AA1 plays a significant role in synaptic homeostasis and protein pathology related to microglia function, and it is among the key factors that could aggravate the synaptic pathology [75]. While HSP90AA1 down-regulation has been shown to reduce microglial activation and Aβ clearance in AD [75], HSP90AA1 has been reported to be up-regulated in PD and related to synaptic decline [20,48]. HSPA1A plays an important role in the degradation of accumulated Parkin [76] and has a key role in the ubiquitin–proteasome mechanism that is directly associated with the disease [77]. HSPA1A up-regulation has been reported in the substantia nigra, which we expanded to the cortical level, but also in the blood at the peripheral level [21]. DNAJA4 is reported to be implicated in neurodegeneration-related protein aggregation [78].

Our study does not go without limitations. While our study’s novelty is unique reporting on transcriptomic changes in fresh cortical biopsies in living patients, it also has some technical limitations related to confounding batch effects, unequal cohort size, and different cell counts. Therefore, these factors were carefully considered when planning and implementing the bioinformatic downstream analysis. For instance, batch correction accounts for technical confounders like different runs and donors, and data integration prevents batches and groups to be dominating and ensures that the detected clusters reflect biological relevance; differential expression through pseudobulking, followed by DESeq2, directly corrects for sample size differences, i.e., avoiding cell count inflation and model sample-level variance. Overall, our approach mitigates unequal representation while ensuring biological representation and not technical or size artifacts. Further, the control group included neuroinflammatory and non- inflammatory conditions. The inflammatory profile found in the PDb diverged from these patients, suggesting disease specificity rather than cohort-wise bias. However, future studies, including larger and stratified cohorts, may further refine these signatures. We explored the possibility of including healthy control samples from biobanks or previously published studies; however, there was a lack of availability of fresh samples from the same region, as our PDb and postmortem samples would not be comparable due to transcriptional changes directly related to the death process. Moreover, scRNA-seq methods have inherent limitations related to dropout events and technical noise. Network and enrichment analyses (WGCNA, GSEA, PANTHER, enrichR, and STRING) are based on previously existing gene annotations and may miss novel or context-specific pathways. Additionally, statistical controlling for false positives may increase false negatives, potentially overlooking subtle but biologically relevant signals. Finally, the microwell SCOPE-chip method (Singleron Biotechnologies) is less effective for human neurons because of their larger size and vulnerability to membrane damage during mechanical and enzymatic dissociation. Both glial cells and neurons engage in a dynamic, bidirectional exchange of signals that shapes cortical oscillations, which are detectable in electroencephalographic (EEG) recordings [79]. Therefore, although glial cells are more suitable to study developments in neuroinflammation and neurodegeneration based on their role as key modulators of immune responses and homeostasis in the CNS, future studies may directly target neurons, e.g., using single-nuclei sequencing, to enrich our interpretations.

Our findings highlight cell type-specific associations between cortical gene expression and electrophysiological features in PD, primarily exploring major glial cell types. Future studies focusing on replicating these findings will be crucial to confirm the extent of this coupling within neural populations. Additionally, back-translating our observed associations into animal models or in vitro experimental conditions represents a necessary step to further evaluate the therapeutic potential of the identified candidate genes or clarifying their mechanistic roles in the transition from healthy to diseased brain states. In particular, experimental studies investigating the effect of P2RX7 antagonism on pathophysiological activity in PD models, as well as targeted manipulation of HSPD1/HSPA9 in glial cells in cell cultures or cell organoids, are essential to allow the assessment of their impact on brain activity and behavioral/clinical outputs.

In conclusion, our findings establish a molecular basis for immunometabolism dysregulation as a central mechanism underlying the widely known electropathophysiological hallmarks of motor symptoms of PD. By directly linking glial transcriptomic alterations in fresh cortical tissue to pathophysiological brain activity, we provide novel evidence that bridges molecular and electrophysiological modalities in living patients. Within these cell-specific enriched metabolic pathways and depleted immune pathways, key genes are at the interface of the inflammasome and can therefore be implemented as biomarkers for patient stratification or as targets for immunomodulatory therapies. These findings not only deepen the understanding of glial involvement in PD but also offer a mechanistic rationale for targeting glial immunometabolic dysfunction as a strategy to modulate abnormal oscillatory activity. Furthermore, this work provides a framework for back-translating oscillation-linked molecular signatures identified in humans into preclinical models, allowing experimental validation of candidate genes like P2RX7, HSPD1, and PRKCB in relation to disease mechanisms and treatment response.

## Methods

### Ethics

All participants provided written informed consent prior to inclusion in the study. The research adhered to the principles outlined in the Declaration of Helsinki and received approval from the local Ethics Committee 837.208.17 (11042). No compensation was offered to the participants.

### Participant selection and description

The electrophysiological study included 91 patients with PD (mean age ± standard deviation: 61.70 ± 11.51 years, 19 females) and 38 healthy controls (mean age: 61.84 ± 9.53 years, 19 females). PD patients and healthy control volunteers were enrolled at the University Medical Center of the Johannes Gutenberg University Mainz. EEG recordings were performed using a 256-channel HydroCel Geodesic Sensor Net system (EGI Netstation, Eugene), referenced to Cz and sampled at 1,000 Hz. Participants underwent EEG measurements, including 5-min resting state, while seated in a comfortable, slightly reclined position with both forearms supported by armrests. They were instructed to keep their eyes closed, move as little as possible, let their mind wander (i.e., not think of something specific), and not fall asleep.

For both EEG and the scRNA studies, patients were clinically evaluated by a movement disorders specialist at the Department of Neurology, University Medical Center of the Johannes Gutenberg University Mainz. Before study enrollment, all patients receiving DBS underwent comprehensive clinical screening and fulfilled all eligibility requirements for DBS [80]. Additionally, a clinical neuropsychologist conducted assessments to rule out cognitive impairment, >24 points in MoCA (Montreal Cognitive Assessment) and >138 for Mattis Dementia Rating Scale. PD patients in the scRNA study had a clinically confirmed diagnosis of PD according to the UK Parkinson’s Disease Society Brain Bank criteria; non-PD patients were selected on the basis of not having a main neurodegenerative or metabolic disease. All patients (PD and non-PD) enrolled for biopsy extraction were additionally screened by multiple physicians and neurosurgeons and participated voluntarily. No compensation was given. DLPFC samples, weighing 50 to 100 mg, were collected from beneath the skull borehole during DBS electrode implantation in 14 patients (mean age: 57.79 ± 15.57 years, 9 females). This included 9 PD patients (mean age: 57.77 ± 15.3 years; mean disease duration: 10.1 ± 5.3 years) and 5 non-PD individuals (mean age: 57.8 ± 16.2 years; mean disease duration: 13.4 ± 5.6 years).

### Sample processing

DLPFC samples were taken during the DBS surgery by the neurosurgeon and immediately placed into sterile 50-ml centrifuge tubes with Hanks’ balanced salt solution (HBSS) and transferred on ice to the service Laboratory. The samples were independently washed an additional 3 times in HBSS to remove blood and stored immediately in GEXSCOPE tissue preservation solution (Singleron Biotechnologies) at 4 °C. Samples were processed within 24 h using the manufacturer’s protocols for cDNA capture, quality control, library formation, and index hybridization. All brain samples met quality standards and yielded sufficient material for sequencing. Sequencing was outsourced and performed on NovaSeq 600 sequencer. Due to the mechanical and chemical digestion process, the microwell SCOPE-chip method (Singleron Biotechnologies) is less effective for human neurons because of their larger size and vulnerability to membrane damage.

### Sequencing data processing

Raw reads were processed with CeleScope (v1.8.1, Singleron Biotechnologies) using the GRCh38 human genome as a reference. Quality control and downstream analysis were performed in Seurat (v4.3.0) [81], with filtering for cells based on detected genes, UMIs (unique molecular identifiers), and mitochondrial counts (>10%).

Batch effects were harmonized using Seurat’s integration workflow, employing “FindIntegrationAnchors” and “IntegrateData” [81]. FindIntegrationAnchors() uses Canonical Correlation Analysis as the default integration method with the following default values (2,000 features, normalization.method = “LogNormalize”, scale = TRUE, reduction = “cca”, dims = 1:30).

Standard single-cell RNA-seq workflows were applied, including principal components analysis (PCA), clustering, and t-stochastic neighborhood embedding (t-SNE) with a resolution of 0.5. The resolution was deepened in progressive iterations until clear distinction between the vascular cell types appearing as different clusters. Cell clusters were manually annotated using known markers and public databases [82], and collapsed where appropriate to reflect a specific cellular level.

Differential gene expression between PD and non-PD groups was assessed using the pseudobulk method for each cell type of interest, followed by DESeq2 (v1.34.0) [83]. Gene set enrichment analysis (GSEA) was performed using GO terms for biological processes (BP) via clusterProfiler (v4.2.2), and the results were visualized using enrichplot [84].

Weighted gene correlation networks (WGCNA) were constructed per cell type, using an unsigned network with soft power and hierarchical clustering [85]. Modules were created (“mergeCutHeight” = 0.4, “minModuleSize” = 100) and functionally profiled using GO terms (BP and MF) with PANTHER [86].

Overlapping genes and terms from GSEA and WGCNA were highlighted with ggvenn and visualized using Ridgeplot (ggplot2). Overlapping genes were further entered into the STRING database to search for potential functional protein–protein interactions [87].

### EEG data processing

EEG data were processed in MATLAB (R2019b, Mathworks) using the FieldTrip toolbox (v20220310)[88]. No subject was discarded due to low quality or incomplete data. Channels above the nasion-Oz line were included. Preprocessing steps involved re-referencing to a common grand average (“ft_preprocessing”), resampling to 250 Hz (“ft_resampledata”), and segmenting the data into 4-s epochs with 50% overlap. Data were detrended, filtered (high pass: 1 Hz, low pass: 95 Hz, band stop: 47 to 53 Hz), and further segmented into 1-s nonoverlapping windows to exclude noisy channels and segments (“ft_rejectvisual”). Independent component analysis removed artifacts such as muscle activity, eye blinks, and eye movements. Rejected channels were interpolated using weighted averages (“ft_channelrepair”).

Afterward, we performed the multitaper frequency transformation using “ft_freqanalysis” with discrete prolate spheroidal sequences and a frequency smoothing of 7 Hz for frequencies ranging from 1 to 100 Hz in 1-Hz steps across the 1-s-long segments to analyze the spectral features of the data. Average beta band power for each participant was calculated as the mean power of frequencies between 13 and 35 Hz and 89 channels of interest covering the fronto-central region. Narrowband gamma power for each participant was first normalized by the average power across channels between 35 and 100 Hz and then calculated as the mean power of frequencies between 60 and 80 Hz and the channels of interest.

To investigate cross-frequency coupling, the beta–gamma phase–amplitude coupling was calculated as the modulation index [89] with the “Matlab toolbox for estimating phase–amplitude coupling” (find_pac_shf_fdr: frequency for beta phase and frequency for gamma amplitude, https://data.mrc.ox.ac.uk/data-set/matlab-toolbox-estimating-phase-amplitude-coupling). The modulation index was then averaged within each subject across the fronto-central channels and frequencies of interest (beta: 13 to 35 Hz; narrow gamma band: 60 to 80 Hz).

### Statistical analysis

#### Sample sizes were not predetermined statistically

EEG data analyses were conducted in FieldTrip (https://www.fieldtriptoolbox.org/), testing group differences (PD, PDb, and HC) in beta power, narrow gamma power, and PAC (https://github.com/sccn/PACTools) using one-sided *t* tests based on prior hypotheses of increased beta and reduced gamma activity, as well as increased PAC [2,4,11,13].

Statistical analyses on scRNA-seq data were performed in RStudio (v1.4.1717), using established packages: Seurat, DESeq2, WGCNA, GSEA, PANTHER, and enrichR. Seurat: Log normalization for scaling and variance stabilization; graph-based Louvain algorithm on the PCA-reduced data for clustering. DESeq2: Differential expression analysis with negative binomial modeling; *P* values adjusted using family discovery rate (FDR) correction. GSEA: Uses a Kolmogorov–Smirnov-like test to calculate an enrichment score (ES) for gene sets in a ranked list. Significance is tested by phenotype-based permutation (shuffling sample labels) to create a null distribution. Normalized ES scores were used with FDR correction. FDR correction coupled with pseudobulk-based differential testing (DESeq2) accounts for multiple comparisons and sample size imbalance preserving sensitivity. WGCNA: Constructs gene coexpression networks by calculating pairwise correlations between genes. It uses hierarchical clustering to identify modules of coexpressed genes. Module–trait associations are tested using Pearson’s correlation with phenotypic data. PANTHER and enrichR: Perform overrepresentation analysis using Fisher’s exact test to assess enrichment of gene sets. *P* values are adjusted for multiple testing using FDR correction. Network and enrichment analyses (WGCNA, GSEA, PANTHER, enrichR, and STRING) were conducted depending on specific research questions.

## Data Availability

Raw data generated in this work and any additional information required to re-analyze the data reported are available upon request from the lead contact. This paper does not report original code. All analyses were performed using openly available software and toolboxes. For the single-cell RNA data, available packages (Seurat, DESeq2, WGCNA, GSEA, PANTHER, and enrichR) were used. For the EEG data, Fieldtrip (https://www.fieldtriptoolbox.org/) and the PAC toolbox (https://github.com/sccn/PACTools) were used.
